# Modeling the Impacts of Urban Flood Risk Management on Social Inequality

**DOI:** 10.1029/2020WR029024

**Published:** 2021-05-21

**Authors:** Simon Moulds, Wouter Buytaert, Michael R. Templeton, Ishmael Kanu

**Affiliations:** 1 Department of Civil and Environmental Engineering, Imperial College London, London, UK; 2 Department of Civil Engineering, Fourah Bay College, University of Sierra Leone, Freetown, Sierra Leone

## Abstract

The exposure of urban populations to flooding is highly heterogeneous, with the negative impacts of flooding experienced disproportionately by the poor. In developing countries experiencing rapid urbanization and population growth a key distinction in the urban landscape is between planned development and unplanned, informal development, which often occurs on marginal, flood-prone land. Flood risk management in the context of informality is challenging, and may exacerbate existing social inequalities and entrench poverty. Here, we adapt an existing socio-hydrological model of human-flood interactions to account for a stratified urban society consisting of planned and informal settlements. In the first instance, we use the model to construct four system archetypes based on idealized scenarios of risk reduction and disaster recovery. We then perform a sensitivity analysis to examine the relative importance of the differential values of vulnerability, risk-aversion, and flood awareness in determining the relationship between flood risk management and social inequality. The model results suggest that reducing the vulnerability of informal communities to flooding plays an important role in reducing social inequality and enabling sustainable economic growth, even when the exposure to the flood hazard remains high. Conversely, our model shows that increasing risk aversion may accelerate the decline of informal communities by suppressing economic growth. On this basis, we argue for urban flood risk management which is rooted in pro-poor urban governance and planning agendas which recognize the legitimacy and permanence of informal communities in cities.

## 1 Introduction

The distribution of flood risk in urban areas is highly heterogeneous (Grasham et al., 2019), with the adverse impacts of flooding experienced disproportionately by the poor (Douglas et al., 2008; Filho et al., 2018; Oliver-Smith et al., 2017; van Voorst, 2015). Flood risk is particularly acute in unplanned, informal urban settlements, which are home to more than 1 billion people worldwide (World Health Organization & UN Habitat, 2016). In these communities, weak governance and systemic inequalities have resulted in a lack of effective policy to mitigate and adapt to the elevated flood risk (Grasham et al., 2019). Instead, many approaches to flood risk management have only succeeded in magnifying the flood risk (Douglas et al., 2008), worsening social inequality and entrenching poverty (Amoako, 2016). The rate of urbanization will continue to rise until around 2050 (UN DESA, 2019), with the number of people living informally expected to reach 2 billion (World Health Organization & UN Habitat, 2016). Around 90% of future urbanization will occur in Africa and Asia (UN DESA, 2019), where climate change is likely to increase the urban flood hazard (Dottori et al., 2018). Consequently, there is a need to improve our knowledge about the dynamic relationship between urban flood risk management, inequality and poverty, to inform the development of sustainable and inclusive policies for reducing urban flood risk (Grasham et al., 2019). Here, we address this knowledge gap by developing a system dynamics model for evaluating the impact of alternative approaches to flood risk management on inequality and poverty in stratified urban societies.

Flood risk is a function of the specific characteristics of the flood hazard and the exposure and vulnerability of society to the hazard (Di Baldassarre et al., 2015). Related to flood risk is the concept of resilience, which we define here as “a neighborhood’s capacity to weather crises such as disasters and engage in effective and efficient recovery through coordinated efforts and cooperative activities” (Aldrich, 2012). In developing countries, rapid urbanization and population growth combined with systemic factors such as complex land tenure arrangements, inadequate transportation and weak governance, as well as the legacy of colonialism (Enqvist & Ziervogel, 2019), has led to the proliferation of unplanned urban settlements which lack basic infrastructure (Herslund et al., 2016; Reckien et al., 2017). As these communities are frequently situated on marginal, flood-prone land (Di Baldassarre et al., 2010; Dodman et al., 2019; Few, 2003), residents are exposed to fluvial and pluvial flooding (Douglas et al., 2008), and related hazards such as landslides (Ezeh et al., 2017; Few, 2003; Reckien et al., 2017). A loss of infiltration capacity combined with inadequate drainage infrastructure heightens the severity of the hazard by increasing the amount of surface runoff (Douglas et al., 2008). Cramped and crowded conditions and poor-quality housing increases the vulnerability of residents to flooding (Di Baldassarre et al., 2010; Zevenbergen et al., 2008). In these conditions, flooding can cause physical, economic, and social devastation, including injury and loss of life, homelessness, and loss of income (Douglas, 2017). They are associated with physical and mental health impacts, including Cholera and diarrheal disease (Grasham et al., 2019), post-traumatic stress disorder (Dai et al., 2017), anxiety, and depression (Ahern et al., 2005).

Poverty is a multidimensional phenomenon, which not only stems from a lack of resources, but also comprises a lack of opportunity, physical and economic security, and empowerment (Few, 2003). Ultimately these aspects of poverty stem from social inequality (Kjellstrom et al., 2007), which encompasses the unjust distribution of income, benefits and services among citizens (Sayers et al., 2018), a lack of recognition of social diversity in planning and policy initiatives, and a lack of inclusivity in political decision-making (Islam & Winkel, 2017; Levy, 2018). Urban flood risk management—broadly defined as the implementation of structural and non-structural measures to reduce urban flood risk (Jha et al., 2012)—has emerged as a central issue for urban planning (Amoako et al., 2019; Di Baldassarre et al., 2014; Watson, 2009). However, in many cities experiencing rapid unplanned development, the response of planning authorities to urban flooding has been reactionary and biased toward the priorities and needs of the powerful (e.g., Amoako, 2016; Cobbinah & Darkwah, 2017; Ríos, 2015). The result is urban flood risk management which to some extent either ignores or victimizes the urban poor (Amoako, 2016; van Voorst, 2015), creating a vicious cycle whereby the initial inequality causes the poor to suffer disproportionately from flooding, which in turn deepens the subsequent inequality (Grasham et al., 2019; Islam & Winkel, 2017). Overall, this speaks to the argument that most water crises—including flooding—are in fact the result of a crisis of governance, arising when scientists, policy makers, and managers fail to understand or adequately consider the broader economic and socio-cultural context of water management interventions (Di Baldassarre et al., 2019).

The Sustainable Development Goals (SDGs) of the United Nations 2030 Agenda for Sustainable Development represent an ambitious and comprehensive blueprint to eliminate poverty, reduce inequality, and create a sustainable future for mankind (United Nations, 2015). Meeting the SDGs relating to water security and disaster risk reduction requires an inclusive approach which involves all actors and stakeholders, and recognizes the various ways in which the earth system and society interact (Bleischwitz et al., 2018). However, traditional approaches to water management are characterized by technical solutions which disregard the dynamic feedbacks between natural and socio-technical aspects of coupled human-water systems (Di Baldassarre et al., 2013; Ziervogel et al., 2016), increasing the likelihood of negative unintended consequences (Leong, 2018; Sivapalan et al., 2014). Socio-hydrology seeks to address this deficiency by studying the two-way feedbacks and interactions between human and natural systems (Sivapalan et al., 2012), with a focus on identifying and understanding emergent phenomena arising from water management activities in a variety of contexts around the world (Konar et al., 2019). Its holistic view of the water system means that socio-hydrology has been identified as having an important role to play in informing water management policies which address the SDGs (Di Baldassarre et al., 2019).

Modeling has emerged as an important mode of inquiry in socio-hydrology which has yielded valuable insights into the co-evolution of flood risk and society (Di Baldassarre et al., 2013, 2015; Srinivasan et al., 2016). However, existing socio-hydrological models have critical shortcomings which limit their capacity to address many of the challenges associated with sustainable development (Blair & Buytaert, 2016; Di Baldassarre et al., 2019). A fundamental weakness of current models is the formulation of urban societies as homogeneous entities which interact uniformly with the water cycle (e.g., Di Baldassarre et al., 2013; Garcia et al., 2016). This simplifying assumption prevents models from considering important within-city heterogeneities (Di Baldassarre et al., 2013), including the uneven distribution of exposure and vulnerability to natural hazards among urban residents; the subjective determination of tolerable risk levels by individuals (Johnson, 2018); and the differential availability of mitigation and adaptation options across society (Amoako, 2018). The resulting models are ill-equipped to provide insight into the relationship between urban water management and social inequality, with a likely consequence that models fail to capture important system behaviors (Di Baldassarre et al., 2019). Given the prominence of reducing inequality to the SDGs, which are premised on the notion of “leaving no-one behind” (United Nations, 2015), there is a need to advance the current generation of socio-hydrological models to explicitly consider social heterogeneity if they are to provide a meaningful contribution to the sustainable development agenda.

Here, we put forward a novel approach to modeling feedbacks between human and natural systems which explicitly accounts for social heterogeneity in the context of urban flood risk management. While the link between flood risk and inequality is apparent in developed and developing cities around the world (e.g., Sayers et al., 2018; Walker & Burningham, 2011), we focus here on rapidly urbanizing cities in low and middle income countries where informal development is a pervasive phenomenon, representing a fundamentally different challenge to flood risk management than those encountered in high income countries with lower rates of urbanization. We adapt an existing socio-hydrological model of floodplain dynamics (Di Baldassarre et al., 2013; Viglione et al., 2014) to account for a stratified urban society comprised of formal and informal settlements. With this conceptualization, we account for within-city heterogeneities in the distribution of flood risk, risk tolerance, and flood risk management. We develop a hypothetical case study consisting of a city divided into planned and unplanned settlements, and use the model to explore the dynamics between these two sections of society, and the co-evolution of the society as a whole, under four scenarios of flood risk management. The study is structured as follows. We first provide an overview of the current literature on the relationship between urban flood risk management and social inequality. This review informs our conceptual model, which is presented in the proceeding section. We describe the case study and four scenarios, and present the model results. Lastly, we critically review our findings, draw conclusions from the study, and highlight future research priorities.

## 2 Inequality and Urban Flood Risk Management

Here we assess the pathways through which urban planning and flood risk management may positively or negatively influence within-city inequality, drawing evidence from cities around the world. Our objective is not to provide an exhaustive review of the literature, but rather to draw out common themes in the interaction between urban flood risk management and social inequality which can be incorporated in our conceptual model.

Urban flood risk management activities may ignore informal settlements or target them with measures such as forcible evictions and site clearance. Based on evidence from Accra, Ghana, Amoako (2016) argues that the vulnerability and exposure of informal urban settlements to flood hazards is to a large extent shaped to by the posture and activities of the state and its urban governance institutions. Amoako (2016) identifies the positioning of urban authorities on a continuum between “brutal presence,” where residents of informal settlements are penalized through strict planning regulations, forced evictions, and demolition, and “convenient absence,” where the state absolves itself of responsibility for planning and the provision of infrastructure. Both extremes are associated with elevated flood risk, but for different reasons. In the first case, the use of aggressive actions to clear informal settlements fails to address the underlying factors driving this form of urbanization and simply displaces the perceived problem to other parts of the city (Amoako, 2016). Often, these locations may render the occupants even more disadvantaged in terms of accessing the labor market and environmental conditions (Ezeh et al., 2017). Moreover, enforced relocation may erode the social capital within and between social groups and, in doing so, reduce resilience (Aldrich, 2012; Mitra et al., 2017). This is evident in Accra, Ghana, where successive actions of authorities to clear informal developments across the city have only served to expand the number of people living in the slums of Old Faddama and Agbogbloshie, located on the banks of the flood-prone and highly polluted Korle Lagoon (Amoako, 2016).

The “brutal presence” position described by Amoako (2016) goes hand in hand with that of “convenient absence,” since the eviction or demolition of one settlement tends to displace its former residents to other such neighbourhoods, usually with the tacit approval of those carrying out the eviction. Moreover, the ensuing animosity between the authorities and residents undermines the legitimacy of the state to govern in informal settlements (i.e., the social contract), promoting a vicious cycle of inaction (Mitra et al., 2017). In interviews conducted by Amoako (2016), one official of the Accra Metropolitan Assembly Town and Country Planning Department observed that the initial settlers of Old Faddama in the early 1980s were ignored by city authorities since the buildings there “were temporary and that they (the residents) will go away due to the lack of basic household infrastructure and services.” However, neglect of Old Fadama by the city authorities led to its continuous growth through the 1990s, mainly as the result of rural-urban migration and migration to Accra by those fleeing an ethnic conflict in northern Ghana (Amoako, 2016). The emergence of an electronic waste hub on the banks of the Odaw River near Old Fadama and Agbogbloshie has provided economic opportunities and, combined with the availability of affordable low-income housing, has fueled the rapid expansion of the two settlements since the turn of the millenium (Amoako et al., 2019). Elsewhere in Jakarta, Indonesia, city authorities absolve themselves of responsibility for flood risk management in informal settlements by blaming residents for the elevated risk, citing the construction of housing on flood-prone land and the disposal of waste in watercourses (van Voorst, 2015).

The absence of city authorities from informal settlements is reflected in the reporting of disasters. In El Colli, Mexico, Gran Castro and Ramos De Robles (2019) found that residents identified flooding as the most important threat to their community based on their recent experience of flood damage, even though no such flood events were recorded in official statistics. In many developing countries small and medium disasters are seldom reported (Bull-Kamanga et al., 2003), even though cumulatively they may account for more casualties than large-scale disasters (Osuteye et al., 2017). In Accra, the livelihoods of many inhabitants center on petty trading and small-scale commerce, which are frequently disrupted by small-scale floods and other disasters (Douglas et al., 2008; Satterthwaite & Bartlett, 2017). The ensuing losses may significantly reduce the ability of households to buy food and pay essential bills, including those for healthcare and education (Douglas et al., 2008). At the same time, the decision to reside in hazardous areas is itself often driven by socio-economic pressures (Johnson, 2018), with informal settlements usually situated close to potential job opportunities (Ezeh et al., 2017). Hence, the growth of informal settlements may be seen as a trade-off between poverty and risk, whereby the urban poor accept an increase in exposure to multiple hazards in order to address the immediate concern of living in poverty (Ezzati et al., 2018; Johnson, 2018). The threshold at which risk becomes intolerable risk varies according to circumstance and strongly influences decisions on how best to approach risk management (Johnson, 2018). A failure to understand the risk perceptions of informal communities—which begins with a lack of reporting of disasters—has tended to promote top-down, technocratic solutions to urban flood risk management which do not align with the needs and priorities of the urban poor (Ziervogel et al., 2016).

In the absence of the state, adaptation to flooding in informal communities is often driven by bottom-up, community-based activities. Such actions have been observed in cities around the world. In Accra, Amoako (2018) studied three communities—Glefe, Agbogbloshie, and Old Fadama—which experience annual flooding, observing that despite poor quality housing and environmental health conditions, the area and population of these communities continued to expand. They (Amoako, 2018) identified a range of adaptation measures adopted by the communities, including restructuring buildings, constructing and maintaining drainage channels, formulating evacuation plans and establishing safe havens. In Visakhapatnam, India, Jain and Bazaz (2020) found that people who had lived for more than five years in locations deemed untenable by city authorities due to the elevated disaster risk had in fact put in place adaptation strategies to reduce risk to a level which was acceptable to the community. This highlights the potential for conflict between city authorities and communities arising from the discrepancy in risk perceptions, and demonstrates the importance of including the views of the community in disaster risk reduction plans (Jain & Bazaz, 2020; Johnson, 2018; Ziervogel et al., 2016). Flooding may also induce changes in governance structures to ensure the response to flood events is more effective. An example of this was seen in Ayutthaya, Thailand, where Ng (2016) observed that an ineffective, reactionary response by the regional government to extensive flooding in 2011 led to further decentralization of flood governance to the local level. Nevertheless, while there is broad agreement that decentralization in disaster risk management has the potential to be a positive step (Ziervogel et al., 2016), it can only be effective if the resulting governance structures have the capacity and financial resources to exercise power (Adelekan et al., 2015; Garschagen, 2016; van Voorst, 2016). Moreover, while neighborhood and household-level adaptation measures may provide some benefit residents of informal settlements (Amoako, 2018), they do not address the systemic causes of elevated flood risk (Dodman et al., 2019; Douglas, 2017).

Many attempts to manage the process of urban growth may exacerbate flood risk for the poor (Pelling & Garschagen, 2019; Weinstein et al., 2019). Rapid urbanization in Asia, Africa and Latin America has led to the resurgence of new towns as a planning strategy to manage urban growth and alleviate pressure on municipal services (Ríos, 2015; Rumbach, 2014). While such towns offer several advantages to planning authorities, they are often situated on marginal, hazard-prone land. In Kolkata, India, Salt Lake has undergone extensive development since the 1970s to transform a former swamp into a residential neighborhood with high-quality housing and flood protection infrastructure (Rumbach, 2017). However, those who serve the wealthy residents of Salt Lake, unable to afford housing in the planned settlement and lacking access to suitable public transport, instead live on the outskirts of the new development in informal settlements where flooding and waterlogging is common (Rumbach, 2017). A similar phenomenon is observed in Buenos Aires, where urban policies skewed toward private interests have resulted in flood risk management activities which aim to raise the value of land for the construction of gated communities, forcing the informal communities which previously occupied the land to move to ever more hazardous locations (Ríos, 2015). Facing rapid urbanization, natural hazards and the effects of climate change, planning authorities and government agencies are increasingly moving people away from hazardous locations (Johnson, 2018). However, even when resettlement is successful at reducing flood risk, it may not succeed in the wider aim of reducing poverty (Jain & Bazaz, 2020; Mitra et al., 2017), leading many development experts to warn against resettlement for urban disaster risk reduction in all but the most extreme cases (Johnson, 2018). Yet this advice often conflicts with the development priorities of national and municipal governments who are keen to attract foreign investment in their cities (Watson, 2014).

From the above discussion, we identify some key themes relating to flood risk management and inequality in the context of rapidly urbanizing cities. Driven by a need to escape poverty, residents of informal settlements are typically prepared to accept a greater amount of risk in order to access economic opportunities, leading to settlements which are highly exposed to flooding and other hazards. The response of city authorities in relation to flood risk management in these locations falls on a continuum between ignorance and hostility. On the one hand, hostile governments pursue policies such as forcible evictions and resettlements that erode social capital and may only succeed in displacing inhabitants to more hazardous locations. Conversely, ignorance of informal settlements leads to inadequate infrastructure and services, insecure tenure, and an inability to enact positive change. These actions are intrinsically linked with urban planning agendas. Despite inadequate planning responses to the elevated flood risk, residents of informal settlements are capable of adaptation and mitigation activities to reduce flood risk to levels which are tolerable at the community level. In the following section, we translate these observations into a quantitative modeling framework to simulate the co-evolution of flood risk in formal and informal communities, to further understand the impact of flood risk management on social inequality.

## 3 Conceptual Model

The starting point for our conceptual model is that presented by Di Baldassarre et al. (2013). This model considers a homogeneous society that begins to develop a flood prone region to exploit the economic benefits associated with living close to the river. The growth of the society is suddenly curtailed by the occurrence of a flood event, resulting in an increased awareness within the community of the flood risk. This gives rise to a flood risk management response by the society, which comprises either the construction of physical protection infrastructure (“technological society”), or moving away from the floodplain (“green society”). The response of the society causes feedbacks to the rest of the system which determine the future flood risk. For the green society, moving away from the floodplain reduces its exposure to catastrophic flooding (the “adaptation effect”), but causes a decline in the rate of economic growth as it can no longer fully exploit the benefits of being close to the river. In the technological society, the capital investment required to build flood protection immediately depletes the resources of the society. Meanwhile the flood protection itself may increase the magnitude of flood peaks by changing the flood attenuation and conveyancing properties of the river system (Di Baldassarre et al., 2009; Heine & Pinter, 2012; Remo et al., 2012). Thus, while the flood protection reduces the number of flood events the society experiences, it also results in the decay of flood risk awareness, inducing a gradual shift toward the river to take advantage of the economic benefits. Eventually, a flood event occurs which exceeds the current protection level, causing the society to suffer catastrophic damage (the “levee effect”).

**Figure 1:**
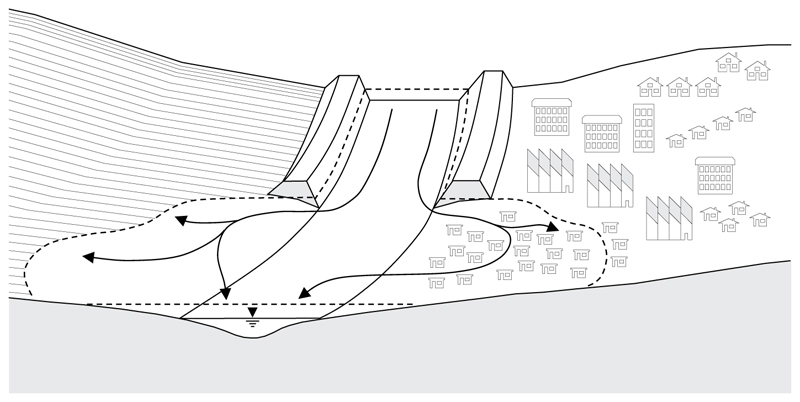
Schematic representation of stratified society, in which part of the society benefits from flood protection while another part does not (adapted from Di Baldassarre et al., 2015).

To explore the impact of flood risk management on social inequality we have adapted the conceptualization of Di Baldassarre et al. (2013) to consider a stratified society consisting of planned and unplanned settlements, with varying levels of exposure and vulnerability to a flood hazard, different levels of risk tolerance, and different constraints on flood risk management (Figure 1). We chose this version of the model, as opposed to its successor (i.e., Di Baldassarre et al., 2015), due to its use of economic prosperity as the main state variable to represent the impacts of flooding on society. This enabled us to effectively represent disparities in economic opportunities between formal and informal communities. In addition, the inclusion of a state variable to represent the distance from the flood plain allows us to parameterize the model such that disparities in the level of tolerable risk could be taken into account. In contrast, the later version effectively replaces the terms representing wealth and distance with population density. While this confers some advantages and results in a simpler model structure, without substantial modifications we found it to be insufficient for our purposes.

In our model, we conceptualize a city authority which is responsible for flood risk management in the planned settlement, but which must also decide how to manage flood risk in the unplanned settlement where the exposure and vulnerability of residents to flooding is greater. We link the two communities through a society awareness term, which conceptualizes the level of awareness of the planned settlement toward the plight of residents in the unplanned settlement. Based on this, the planned settlement—through the city authority—may choose to share a proportion of its wealth and power with the unplanned settlement, enabling it to recover from flood events and potentially install flood protection. The model equations outlined below are based on those presented in Viglione et al. (2014), who expressed the original model equations of Di Baldassarre et al. (2013) in nondimensional form.

### 3.1 Hydrological Forcing

The model is driven by a time series of peak-over threshold of natural high water levels, *W*(*t*), which is represented as a marked point process in which the number of flood events per unit time is assumed to follow a Poisson distribution (Viglione et al., 2014). Time is scaled by the mean time between flood events such that it becomes nondimensional. Thus, for *t* > 0, the number of flood events in the interval [0, *t*] follows a Poisson distribution with mean equal to *t*, and the sequence of times between flood events is expressed as

(1) $$P(T\leq t)=1-e^{-t}.$$

The magnitude of the flood peaks is modeled as a generalized Pareto distribution with minimum equal to zero and unit mean, such that

(2) $$P(W\leq w)=1-{\left(1-\frac{\theta }{1+\theta }w\right)}^{\frac{1}{\theta }}$$

where *θ* is the shape parameter of the Pareto distribution. As in Viglione et al. (2014), we set *θ* = 0.28 such that the modeled high water levels cannot exceed 4.57 in nondimensional form.

### 3.2 Coupled Human-Flood System

We model the settlements as separate human-flood systems, which are dynamically linked through a wealth redistribution term. Each system is parameterized separately according to its assumed physical and socioeconomic characteristics. The dynamic human-flood system is modeled with five differential equations:

(3) $$\{\begin{array}{lll} \frac{dG}{dt}=\rho_{E}(1-D)G+T-\Delta (\Upsilon (t))\left(FG+\gamma_{E}R\sqrt{G}\right) & \text{Economy} & \left(3a\right)\\ \frac{dD}{dt}=\left(M-\frac{D}{\lambda_{P}}\right)\frac{\phi }{\sqrt{G}} & \text{Politics} & \text{(3b)}\\ \frac{dH}{dt}=\Delta (\Upsilon (t))R-\kappa_{T}H & \text{Technology} & \text{(3c)}\\ \frac{dM}{dt}=\Delta (\Upsilon (t))S-\mu_{S}M & \text{Community} & \text{(3d)}\\ \frac{dM^{\prime }}{dt}=\Delta (\Upsilon (t))S^{\prime }-\mu_{S}^{\prime }M^{\prime } & \text{Society} & \text{(3e)} \end{array}$$

As in the notation adopted elsewhere, model variables are capitalized (for clarity, we do not explicitly show that these vary in time *t*—for example, *G* could be written *G*(*t*)), and parameters are denoted using Greek letters. The expression Δ(*𝛶*(*t*)) is a non-periodic Dirac comb which is always zero except when flooding occurs (𝚼(*t*) = 0), at which point it has a value of +∞ with unit integral. Model variables change in time, and are computed separately for each settlement included in the simulation. The main difference between the above set of equations and those described by Di Baldassarre et al. (2013) is the reformulation of the *Society* equation to account for the awareness of communities to flooding occurring in other parts of the city, and the addition of a Community equation, which models the awareness of communities to flooding in their own neighborhood. These will be explained further in due course.

The variation in wealth, *G*, as the settlement experiences and responds to flooding is modeled using Equation 3a—the *Economy* equation. The key departure from the definition included in Viglione et al. (2014) is the addition of a wealth redistribution variable, *T*, which represents the transfer of wealth from the planned settlement to the unplanned settlement:

(4) $$T=(\bar{G}-G)M^{\prime }\tau$$

where $\bar{G}$ is the mean wealth across all settlements, *M′* is the awareness of flood events which occur elsewhere, and *τ* is a parameter which represents the maximum extent by which the wealth gap may be closed following a flood event. For example, if *M*′ = 1 and *τ* = 1, wealth will be redistributed such that there is total equality in wealth. Besides wealth redistribution, Equation 3a states that the variation in wealth is driven on the one hand by economic growth (*ρ_E_*(1 − *D*)*G*), and on the other hand by the shock of experiencing flooding, causing economic damage (FG) and potentially resulting in capital expenditure on flood protection infrastructure $\left(\gamma_{E}R\sqrt{G}\right)$. Parameter *γ_E_* is the unit cost of installing flood protection infrastructure. Economic growth is equal to the maximum economic growth rate experienced at the riverfront (*ρ_E_*), inversely scaled by the distance from the riverfront and multiplied by the current wealth.

The damage experienced by the settlement as a result of high water levels, *W*(*t*), is represented by the variable *F*, which varies between zero (no damage) and one (complete destruction), and is calculated as

(5) $$F=\{\begin{array}{cc} 1-e^{-\frac{W+\xi_{H}H_{-}}{\alpha_{H}D}}, & \text{if}W+\xi_{H}H_{-}<H_{-}\\ 0, & \text{otherwise}. \end{array}$$

This formulation implies that flood damage depends on model state variables representing the current height of flood protection, *H* (*H*_−_ denotes the height of flood protection immediately prior to a flood event), and the distance from the riverfront, *D*. Parameter *ξ_H_* controls the extent to which the water level increases following an increase in the height of the levee. In the original conceptualization of Di Baldassarre et al. (2013), parameter *α_H_* was related to the slope of the floodplain and determined the reduction in expected damage as a society moved further away from the river. Here, as in other applications of the original model (Ciullo et al., 2017; Di Baldassarre et al., 2015), we apply a broader definition of this parameter to include aspects of the built environment that influence people’s vulnerability to flooding, such as the effectiveness of drainage infrastructure and the quality of housing (Amoako, 2018).

The increase in the level of flood protection (e.g., raising levees) following a flood event is modeled as

(6) $$R=\{\begin{array}{ll} \epsilon_{T}\left(W+\xi_{H}H_{-}-H_{-}\right), & \text{if}(F>0)\\ \text{and}\left(FG_{-}>\gamma_{E}R\sqrt{G_{-}}\right)\\ \text{and}\left(G_{-}-FG_{-}>\gamma_{E}R\sqrt{G_{-}}\right)\\ 0, & \text{otherwise}. \end{array}$$

After flooding occurs (*F* > 0), the community makes a decision about whether or not to increase the level of flood protection by evaluating the cost of installing protection $\left(\gamma_{E}R\sqrt{G_{-}}\right)$ against the flood damage which has just been experienced $\left(FG_{-}>\gamma_{E}R\sqrt{G_{-}}\right)$, and their available resources (*G*_−_−*FG*_−_). The amount by which the protection level is increased is the difference between previous high water level and the increase in flood peak induced by the structure (*W* + *ξ_H_H_−_*), and the level of protection immediately prior to the flood event (*H*_−_). Parameter *ϵ_T_* is a safety factor which is used to control the extent to which the flood protection level is increased above the previous high water level.

The change in distance between the settlement and the riverfront over time is modeled using Equation 3b, which is referred to as the *Politics* equation. The rate of change is driven by the economic incentives to move away from the floodplain, considering both the expected damages from future flood events and the opportunities for economic growth, as well as the ability of the settlement to move away. Awareness of flood risk, *M*, plays an important role in the calculation: all else being equal, higher levels of awareness increase the rate at which the settlement moves away from the riverbank. However, the influence of flood awareness on the rate of change is offset by the potential economic benefits of living closer to the river (−*D*/*λ_P_*). Parameter *λ_P_* represents the tradeoff between the desire to maximize economic growth by living as close as possible to the river, against the desire to move away from the riverbank to limit future exposure to flood events. In the original formulation, it represented the critical distance beyond which the community could not grow (Di Baldassarre et al., 2013). Hence, its inverse (i.e., 1/*λ_P_*) is indicative of the amount of risk which the community is willing to endure in order to benefit from opportunities for economic growth (Viglione et al., 2014). The last term in Equation 3b $\left(\phi /\sqrt{G}\right)$ defines the rate at which settlements can shift their center of mass to be closer or further away from the riverfront. This is assumed to be inversely proportional to the representative length of the settlement $\left(\sqrt{G}\right)$, reflecting that larger settlements tend to be harder to move than smaller settlements.

The change in the level of flood protection over time is modeled with Equation 3c, which is called the *Technology* equation. It includes terms that define the installation of flood protection (Δ(𝛶(*t*))*R*), which is governed by the output of Equation 6, and the decline in the level of flood protection (*κ_H_H*), where *κ_H_* is the rate of decay. The level of flood protection is only increased immediately following a flood event, which is modeled by the Dirac comb function.

The variation in community awareness of flood risk over time is represented by Equation 3d, or the *Community* equation. Note that we use the term “society” to the city as a whole—that is, the planned and informal communities together—while “community” is used to refer to the settlements individually. The formulation of Equation 3d is comparable to Equation 3c, as it consists of terms describing the level of community shock immediately following a flood event (Δ(*𝛶*(*t*))*S*), and the decline of flood risk awareness over time (*μ_S_M*), which similarly depends on a rate parameter, *μ_S_*. The shock magnitude varies between zero and one and is closely related to the relative damages of flood events. It is modeled as

(7) $$S=\{\begin{array}{cc} \alpha_{S}F, & \text{if}\;R>0\\ F, & \text{otherwise}. \end{array}$$

After a flood event, the shock magnitude is reduced if additional protection measures are installed (i.e., *R* > 0), or set equal to the relative damage arising from the flood event otherwise. The extent to which the shock is dampened by additional flood protection is controlled by parameter *α_S_*, which may be interpreted as a measure of trust a community places in the protection infrastructure (Viglione et al., 2014). Hence, when *α_S_* = 0 the community has complete trust that the protection infrastructure will prevent any future flood damage, while for *α_S_* = 1 the community maintains its awareness of the flood risk, regardless of the presence of additional protection.

Lastly, the variation in society awareness of flood events which occur elsewhere (that is, flood events which do not directly affect the residents of the community under consideration) is modeled using Equation 3e. It is an analogue of Equation 3d, with *M*′ representing the memory of flood events which occurred in other settlements, and *μ_S_*′ the rate at which the memory or awareness of these events decays. The level of shock *S*′ associated with these flood events is calculated as

(8) $$S^{\prime }=\{\begin{array}{cc} \alpha_{S}^{\prime }F^{\prime }, & \text{if}\;R^{\prime }>0\\ F^{\prime }\text{,} & \text{otherwise}. \end{array}$$

where *α_S_’* represents the extent to which the shock level is moderated by the installation of flood protection in the community which has suffered flooding.

## 4 Model Application

In the first instance, we use the model to develop a set of system archetypes which quantify four approaches to flood risk management and disaster recovery. These are based on the framework of Pelling (2020), who describes alternative post-disaster development pathways based on the intersection of the SDGs and the Sendai Framework for Disaster Risk Reduction (“Sendai Framework,” henceforth). We have translated these pathways to our model by developing a set of model parameterisations which represent the essential characteristics of the accompanying narratives (Pelling, 2020).

**Figure 2:**
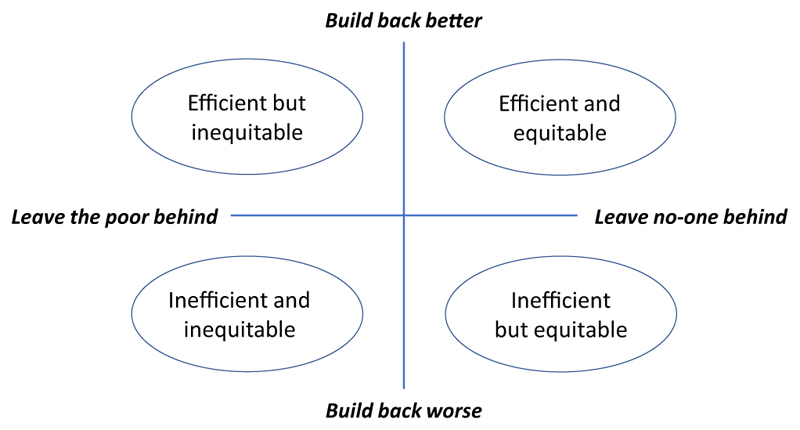
The intersection of the United Nations Sustainable Development Goals, which are premised on “leaving no-one behind,” and the Sendai Framework for Disaster Risk Reduction, which advocates to “build back better” (adapted from Pelling, 2020).

According to Pelling (2020), the SDGs are characterized by their overarching aim of “leaving no one behind”; a deliberate response to criticism of the previous Millenium Development Goals, which inadvertently encouraged governments to focus on raising those just under arbitrary poverty lines to just above, effectively ignoring those in extreme poverty (Pelling & Garschagen, 2019). As a supplementary technical agreement to the SDGs, the Sendai Framework rather focuses on specific measures that governments could take to reduce risk. It’s overarching aim is therefore to “build back better,” and is characterized by a focus on efficiency. These twin aims—”build back better” and “leaving no one behind”—and their opposites, combine to give four possible scenarios, as shown in Figure 2. A description of each scenario is provided in Table 1. For a more detailed description of the four scenarios and additional examples in the context of disaster risk management we refer to Pelling (2020).

While these scenarios are idealized, they provide a useful framework with which to consider the impact of flood risk management on social inequality. In the first part of our analysis, we develop a set of model parameterisations to represent each of the four scenarios, and use these to simulate the co-evolution of the two settlements. We compare the scenarios by looking at the co-evolution of the main state variables in each settlement. The evolution of the society as a whole is considered in terms of the combined wealth and the Gini coefficient (Gini, 1912), a widely used indicator of inequality which measures the distribution of wealth across a population.

The parameter sets corresponding to the four scenarios are shown in Table 2. We consider efficiency solely in terms of economic growth, with *ρ_E_* set to 1 for high efficiency scenarios and 0.5 for low efficiency scenarios. Given that the economic prospects of the unplanned community is contingent upon that of the planned community, economic growth is assumed to be consistent across the society. On the other hand, we consider equity both in terms of the variation in exposure and vulnerability between planned and unplanned settlements, and the different types of flood risk management which are employed by the two communities. We identify two parameters pertaining to urban flood risk which may be expected to vary along lines of wealth and power. First, we use parameter *α_H_* to account for intracity disparities in the vulnerability of communities to flooding, taking into account physical and social factors such as the quality of housing, existence of drainage infrastructure, and access to healthcare. Second, we use the differential value of *λ_P_* to account for the fact that the urban poor tend to exhibit greater risk-taking behavior with respect to location-based risks such as flood risk (e.g., Ezeh et al., 2017; Olthuis et al., 2015; van Voorst, 2015), in order to take advantage of economic and social opportunities (Ezeh et al., 2017). This results in the unplanned community tending to settle closer to the riverfront where the economic opportunities are greater.

Flood risk management is modeled as a combination of “technological” and “green” measures for reducing the risk of flooding (Di Baldassarre et al., 2013). The tendency toward one or other of these approaches is primarily controlled by varying the unit cost of flood protection, *γ_E_* (Di Baldassarre et al., 2013), which we set to 0.005 for both settlements to denote a primarily “technological” approach to flood risk management (Viglione et al., 2014). The inequality of power between the planned and unplanned settlements is modeled by varying the safety factor for raising flood protection, *ϵ_T_*. In the two low equity scenarios we prevent the unplanned settlement from installing flood protection by setting *ϵ_T_* = 0, while in the high equity scenarios we set *ϵ_T_* = 1.1 for both settlements. Another option here would be to set 0 ≤ *ϵ_T_* ≤ 1.1 to account for community-based flood protection measures such as raising homes on stilts (Amoako, 2018). In the high equity scenarios we enable wealth redistribution by setting *τ* = 0.5, which means that when *M*′ = 1 the society commits to reducing the wealth gap by 50%.

In the second stage of the analysis we examine the sensitivity of the model to the differential values of vulnerability, *α_H_*, and appetite for risk, 1/*λ_P_*, which are key indicators of inequality. The purpose of this analysis is to highlight the effectiveness of potential strategies for flood risk management within each of the system archetypes outlined in the first part of the analysis. In each of the scenarios described in Table 2, we vary *α_H_* such that 2 ≤ *α_H_* ≤ 10, and *λ_P_* such that 0.3333 ≤ *λ_P_* ≤ 2.

## 5 Results

### 5.1 System Archetypes

**Figure 3:**
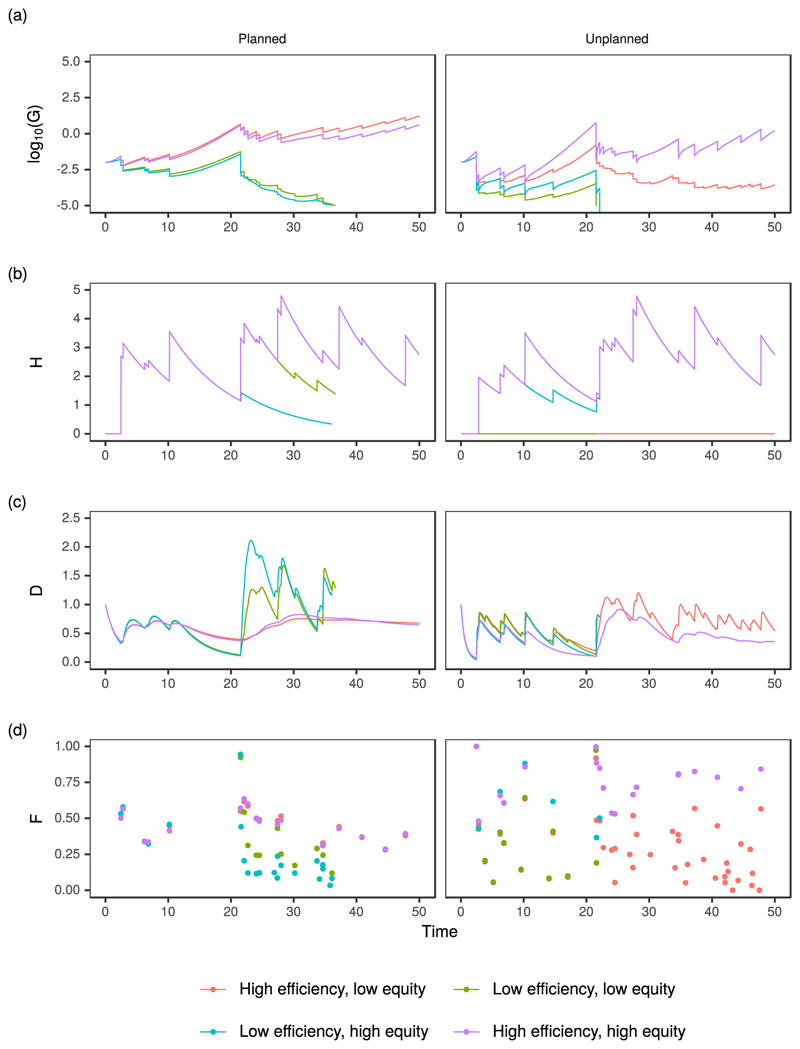
System behavior for each archetype in Table 2 for one possible time series of high water level: (a) community wealth; (b) height of flood protection; (c) distance from the riverbank; (d) relative flood damage. The results are shown separately for the planned and informal settlements.

In this part of the analysis, we model the co-evolution of the planned and unplanned settlements for each of the four scenarios outlined above, using the corresponding parameter sets shown in Table 2 and one possible time series of high water levels. The two low efficiency scenarios result in the long-term decline of both planned and unplanned settlements (Figure 3). For the unplanned settlement the decline is initiated by the large flood event around *t* = 20. In the “low efficiency, low equity” scenario this event causes an immediate collapse, while for the corresponding high equity scenario the collapse is the result of a secondary flood event which occurs shortly after the initial event. The planned settlement survives these flood events, but the damage it experiences triggers a terminal decline in its wealth, which is gradually depleted by successive flood events during the period *t* = [20, 40]. The levee effect is clearly identifiable in the evolution of the planned settlement for the period *t* = [0, 20], during which the planned settlement is able to install flood protection in both high and low equity scenarios. However, during the period of no flooding, *t* = [10, 20], the settlement moves closer to the riverfront while the level of protection gradually declines. A subsequent flood event results in a large amount of damage and provokes the rapid migration away from the floodplain, leading to a marked decline in the economic growth of the settlement which ultimately causes its demise.

In the high efficiency scenarios both planned and unplanned settlements survive over the simulation period. In the planned settlement, there is only a marginal difference in the evolution of the various state variables between the low and high equity scenarios, yet this translates to a large disparity in the corresponding variables of the unplanned settlement. In the low equity scenario, the inability of the unplanned settlement to install flood protection combined with the high economic imperative to reside near to the riverbank leads to frequent flood damage, especially during the interval *t* = [20, 50]. After a flood event, the community initially retreats from the floodplain before quickly returning (Figure 3), driven by the high economic incentive to live close to the riverbank. Such behavior may be interpreted as a temporary migration of the community away from the flooded region until the floodwater has receded and the necessary repairs made.

It arises because of the higher vulnerability of the unplanned settlement (*α_H_* = 5.2) combined with a greater tolerance for risk (1/*λ_P_* = 0.666). Persistent flood damage causes the economic growth of the settlement to stagnate, although the high economic growth rate (*ρ_E_* = 1) ensures it does not fail during the course of the simulation. In the high equity scenario, the ability of the unplanned settlement to install flood protection means that it experiences sustained economic growth during the simulation, punctuated by losses from flood damage. Although both settlements have the same level of flood protection, the relative damages experienced by the unplanned settlement are higher due to it having greater vulnerability (*α_H_* = 5.2), and higher tolerance of risk (*λ_P_* = 1).

**Figure 4:**
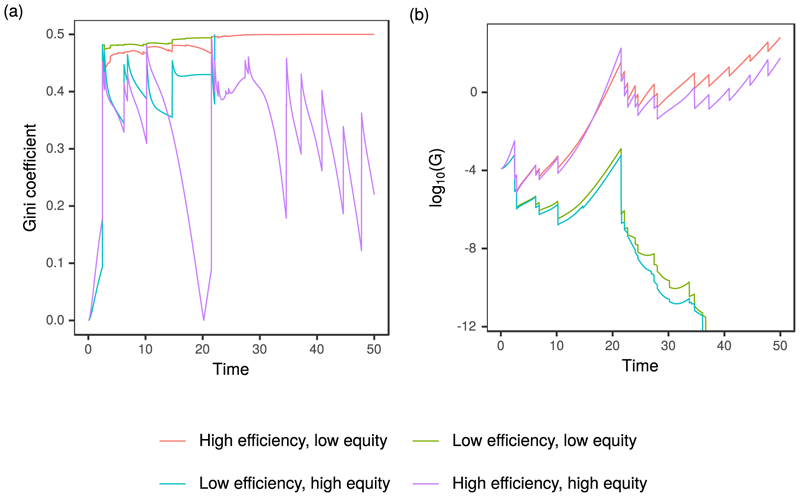
Evolution of the Gini coefficient and combined wealth of the planned and informal communities for each of the four scenarios depicted in Figure 3.

Flood events at the beginning of the simulation cause inequality to rise in all cases (Figure 4), mainly because of the disparity in vulnerability and exposure of the two settlements. In the two high equity scenarios, inequality decreases during dry periods but rises rapidly after flood events. This is due to the differential value of vulnerability (*α_H_*), combined with a greater tolerance of risk among the unplanned community. In the latter half of the “high efficiency, high equity” simulation there is a gradual trend of decreasing inequality, which stems from the ability of the unplanned settlement to install flood protection and protect its economic growth.

### 5.2 Sensitivity Analysis

#### 5.2.1 High Efficiency, Low Equity

In this scenario the parameters representing the vulnerability and risk tolerance of the planned settlement is fixed (*α_H_* = 10 and *λ_P_* = 2, respectively), while for the informal settlement they vary (*α_H_* = 4→10, and *λ_P_* = 0.3333→2). Since in this scenario there is no wealth redistribution (i.e., *τ* = 0), the two settlements evolve independently, with the evolution of the planned settlement the same in each case. At the highest levels of vulnerability and risk aversion (*α_H_* = 4 and *λ_P_* = 2), the wealth of the informal settlement is depleted rapidly as catastrophic flood damage causes the community to move away from flood-prone regions to areas which provide less opportunities for economic growth. For the same level of vulnerability but greater tolerance of risk (*λ_P_* = 0.3333), the unplanned community stays close to the floodplain where the potential for economic growth is highest. While in some cases this attitude results in economic collapse, it is more often the case that the community maintains a tenuous existence which is characterized by periods of economic growth interspersed with catastrophic flood damage. Accordingly, economic growth is not sustainable and the community merely maintains its presence on the floodplain. For high levels of vulnerability (e.g., *α_H_* ≤ 6.4), inequality is consistently high, driven by frequent and severe flood damage in the unplanned settlement.

**Figure 5:**
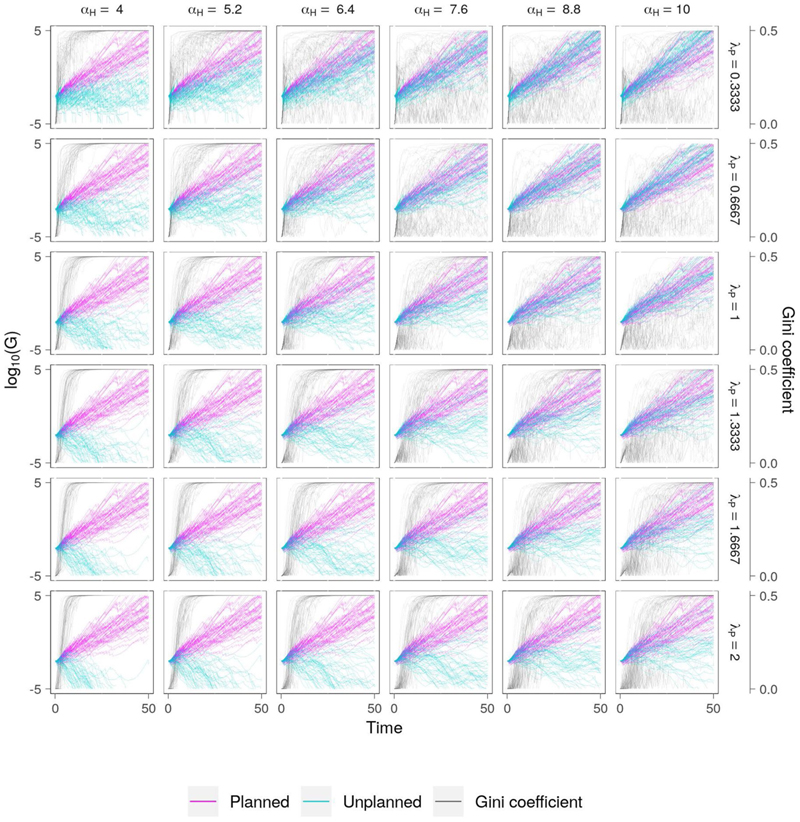
Co-evolution of wealth and social inequality in the planned and informal communities for the “high efficiency, low equity” scenario (Table 2). Parameters relating to vulnerability (*α_H_*) and risk aversion (*λ_P_*) in the informal settlement are varied to investigate model sensitivity and represent potential policy directions. Model simulations in which there is a large difference in wealth result in high social inequality (high Gini coefficient). In this case, the highest social equality is achieved in scenarios when the appetite for risk is high (e.g., *λ_P_* = 0.3333). However, the large spread in Gini coefficient for these simulations indicates that, according to our model, this high-risk approach may exacerbate poverty depending on the evolution of water levels. This outcome is more likely with increasing vulnerability (*α_H_* ↓).

As the vulnerability of the informal settlement reduces (*α_H_* ↑) the relative damage of flood events also declines, allowing the community to sustainably accumulate wealth (Figure 5). However, since the relative flood damage suffered by the unplanned community is always higher than that of the planned settlement due to a lack of flood protection, inequality increases across all water level time series, albeit at a slower rate than for lower levels of vulnerability in the informal settlement.

#### 5.2.2 Low Efficiency, Low Equity

**Figure 6:**
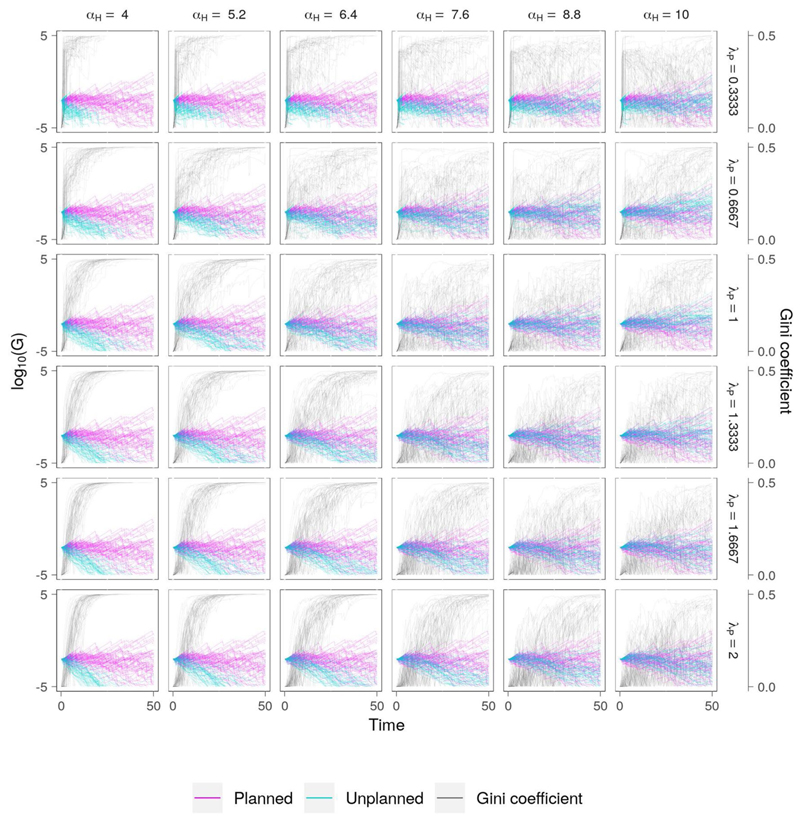
Co-evolution of wealth and social inequality for the “low efficiency, low equity” scenario (Table 2). The only difference between this scenario and the “high efficiency, low equity” scenario is a lower economic growth rate for both communities. This has a large effect on the evolution of the planned settlement, which cannot accumulate enough wealth to develop resilience to flooding. Since it also suppresses the economic benefit of living close to the riverbank, the sensitivity of the model to risk aversion (*λ_P_*) is also reduced. The overall effect is to reduce social inequality but at the expense of economic growth.

This scenario clearly demonstrates the impact of lower economic growth on the ability of both communities to cope with flooding (Figure 6). Nevertheless, some similar patterns emerge. To start with, we again see a rapid gain in inequality when the vulnerability of the unplanned settlement is high. However, unlike in the previous scenario, the settlement typically fails to survive until the end of the simulation, regardless of the appetite for risk. This is because the economic gain associated with living close to the floodplain is much lower as a result of the lower economic growth (*ρ_E_* = 0.5), while the frequency of flooding remains the same. As vulnerability reduces (e.g., *α_H_* ≥ 7.6) the range of inequality seen for the highest appetite for risk (*λ_P_* = 0.3333) also increases, reflecting the lower relative damage experienced by the community, and consequently its increased ability to recover following flood events, combined with the greater economic opportunities afforded by living close to the riverfront.

#### 5.2.3 Low Efficiency, High Equity

**Figure 7:**
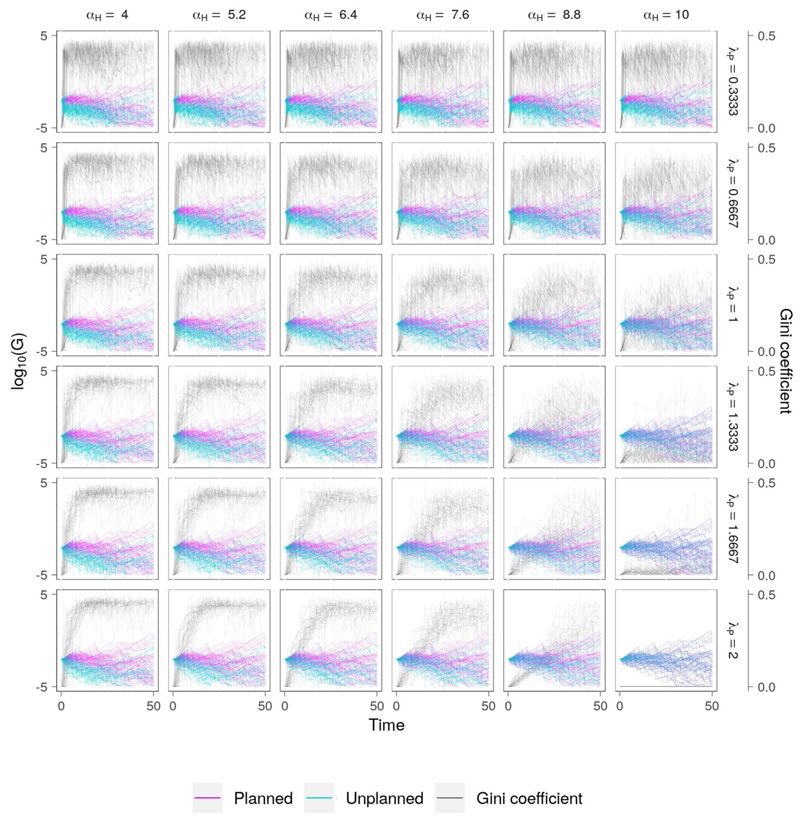
Co-evolution of wealth and social inequality for the “low efficiency, high equity” scenario (Table 2). This differs from the “low efficiency, low equity” scenario by introducing wealth redistribution (*τ* = 0.5), which translates to greater levels of social equity across all combinations of vulnerability and risk aversion. However, despite a commitment to social equality, low economic growth means that neither community can consistently build resilience to flooding, resulting in frequent flood damage and loss of wealth.

Here, the level of disparity in wealth between the two settlements is lower than in the previous scenario (Figure 7), driven by a commitment to equity (*τ* = 0.5). Wealth redistribution has the effect of reducing the ability of the planned community to cope with flooding, which fails more frequently than in the “low efficiency, low equity” scenario. In the majority of cases the wealth of both communities decreases over time. This inefficiency arises because the wealth which is redistributed to the unplanned community is sufficient only to meet the immediate needs of the post-disaster recovery. It may allow the community to continue to exist, but in failing to support effective flood risk management it prevents sustainable economic growth, and the settlement continues to experience flood damage. At the same time, the resources of the planned settlement are increasingly drained by the combined effect of frequent flood events and wealth redistribution.

#### 5.2.4 High Efficiency, High Equity

**Figure 8:**
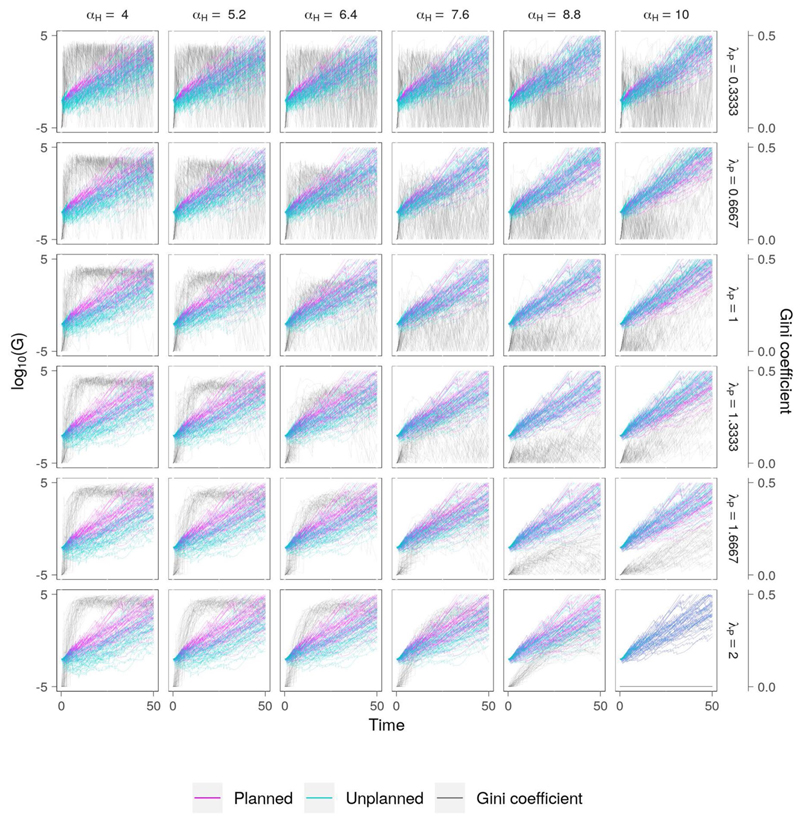
Co-evolution of wealth and social inequality in planned and informal communities for the “high efficiency, high equity” scenario (Table 2), which represents the ideal intersection of the Sustainable Development Goals and the Sendai Framework (Pelling, 2020). Here, the commitment to social equality and relatively high economic growth rate means that the development of the society tends to be inclusive and sustainable, with both settlements experiencing less flood damage. However, this outcome is only achievable when the vulnerabilty of the informal settlement is low (*α_H_* ↑), at moderate levels of risk aversion.

Prioritizing equality (*τ* = 0.5) and efficiency (*ρ_E_* = 1) tends to yield sustained economic growth and lower levels of inequality across the two settlements (Figure 8). As in the previous cases, higher vulnerability is associated with higher inequality. When high vulnerability is combined with a low appetite for risk (e.g., *λ_P_* ≥ 1.3333), flood events early in the simulation causes inequality to rise as the differential values of *α_H_* causes the unplanned community to experience more damage. Across all parameter combinations, the highest levels of inequality in this scenario are seen when the risk-taking attitude of the unplanned community is highest, which results in the settlement experiencing higher relative flood damages than for equivalent scenarios with a lower appetite for risk.

**Figure 9:**
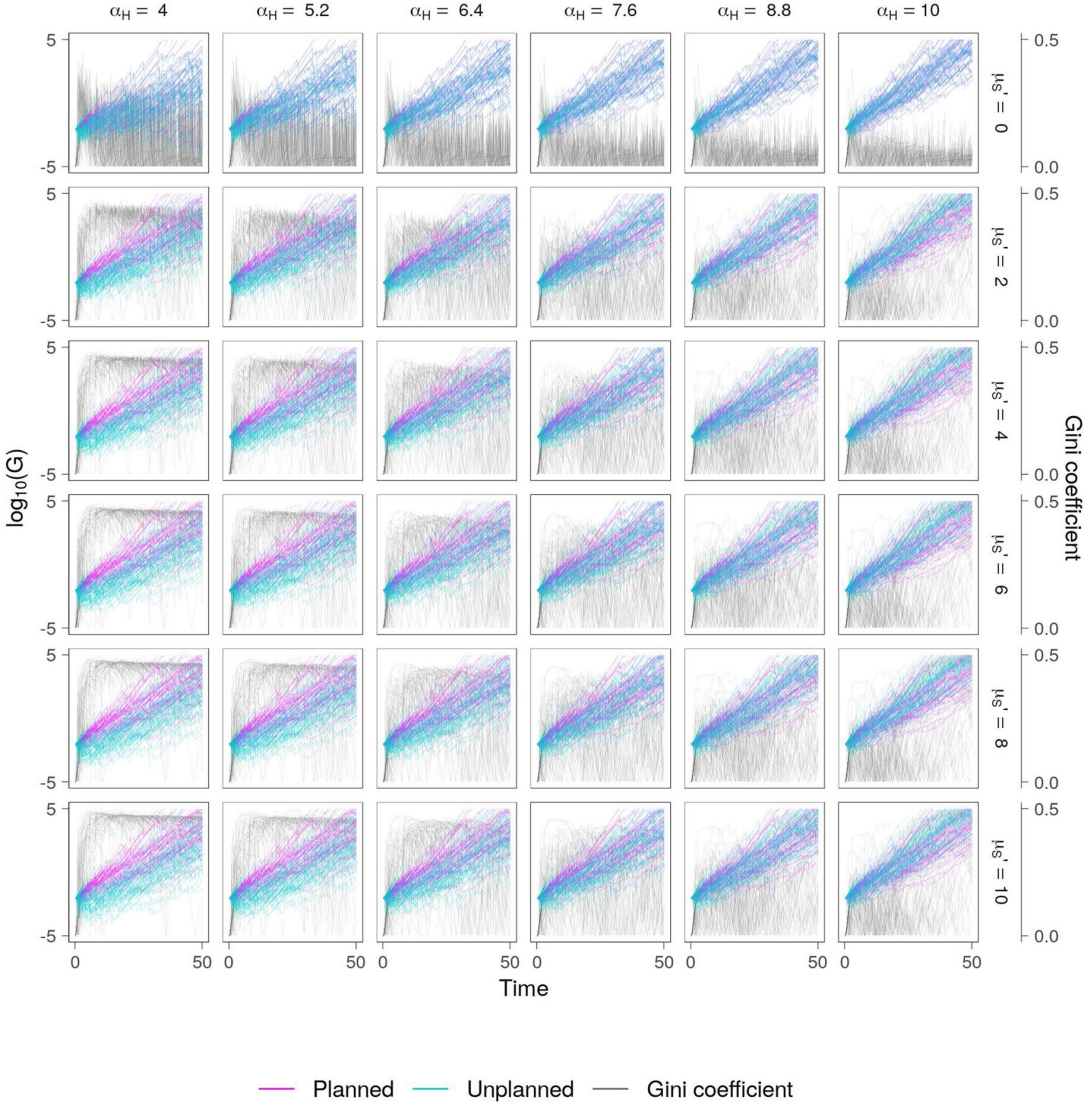
Co-evolution of wealth and inequality in planned and informal communities for the “high efficiency, high equity” scenario, with fixed appetite for risk (*λ_P_* = 0.6667) and the society awareness of flooding is varied. The second row of this plot is the same as that in of Figure 8. Decreasing memory loss rate (*μ_S_′* ↓) increases the tendency toward social equity as the commitment to equality remains high throughout the simulation.

The level of wealth redistribution depends not only on the value of *τ*, but also the awareness of flood events which occur elsewhere, *M*′. The reduction in awareness of these events over time is controlled by parameter *μ_S_*′, where low values translate to a long memory of flood events and high values indicate a short memory. As memory fades, so too does the amount of wealth redistribution. Hence, this formulation may be used to compare the effect of a city authority which is committed to reducing inequality yet reactionary, versus one which is prepared to engage with communities over the longer-term in order to effectively manage the flood risk. To further understand the influence of society memory on the model output, we select the set of scenarios from Figure 8 for which *λ_P_* = 0.6667, and construct a set of parameter combinations in which *μ_S_′* varies from 0 to 10, translating to long and short memory of flood events occurring elsewhere, respectively (Figure 9). Predictably, the highest levels of equality are seen when *μ_S_* = 0, which effectively means that awareness of flooding in other parts of the city does not diminish over time. However, as vulnerability increases (*α_H_* ↓) the variability in social inequality also increases, suggesting that the informal community experiences frequent damage which is repaired at the expense of the planned community.

## 6 Discussion

We have adapted the socio-hydrological model of Di Baldassarre et al. (2013) and Viglione et al. (2014) to simulate the floodplain dynamics of a stratified urban society and investigate the impact of various flood risk management strategies on inequality and flood risk. While the model application is hypothetical, the system dynamics it reveals are consistent with observations from flood-prone settlements around the world (Table 1).

The modeling results reveal potential pathways to reduce flood risk in informal settlements. The sensitivity analysis for the “high efficiency, low equity” scenario (Figure 5 suggests that investing in measures to reduce vulnerability to flooding may have a large effect on the survival and economic prosperity of informal communities. When the risk threshold is high (e.g., *λ_P_* = 0.3333), reducing vulnerability (*α_H_* ↑) generally results in substantial increases in wealth accumulation for the unplanned settlement and a corresponding decrease in social inequality. Although in this case the unplanned community gravitates toward the riverbank despite the inability to install flood protection (*ϵ_T_* = 0), reducing vulnerability tends to have a positive effect on evolution of the settlement. Conversely, policies which either deliberately or inadvertently increase vulnerability are likely to increase social inequality. Again, this phenomenon is often seen in private-sector led urban development such as in Buenos Aires (Ríos, 2015). On the other hand, reducing the threshold of tolerable risk (*λ_P_* ↓) impedes the economic growth of the settlement and increases social inequality, highlighting the fact that marginalized communities must take far greater risks than wealthier communities in order to access economic opportunities.

These outcomes imply two broad policy directions for reducing urban flood risk in informal settlements. On the one hand, policymakers can focus on reducing the vulnerability of existing informal settlements, which in some cases may have a large positive impact on the ability of communities to withstand flooding and grow sustainably (Olthuis et al., 2015). This is the motivation behind many slum upgrading projects, which have successfully reduced flood risk in cities such as Nairobi (Mitra et al., 2017). According to Mitra et al. (2017), successful upgrading projects build the social contract between informal communities and government, enhance social capital within and between social groups, and adopt a multi-sectoral approach to enhancing the urban environment. On the other hand, Olthuis et al. (2015) criticizes many upgrading projects for adopting generic measures which fail to properly consider the specific characteristics of settlements, leading to missed opportunities for reducing flood risk and measures which soon become obselete, contributing to the perception of informal settlements as temporary. As slum upgrading implies that communities continue to reside in the same place, it has parallels with the “technological” approach to flood risk management outlined by Di Baldassarre et al. (2013). However, while some upgrading approaches may be supplemented with large-scale flood protection infrastructure (Olthuis et al., 2015), such measures are not always practicable due to the close proximity of many informal settlements to the river, as well as the considerable expense (Mulligan et al., 2016). Rather, specific interventions to reduce flood risk may include raising homes on stilts, establishing safe havens, and developing community-based early warning systems (Amoako, 2018).

Meanwhile, policies can facilitate the development of less hazardous land which does not deprive residents of economic opportunities and essential services (e.g., schools, health facilities). This option may be need to be accompanied by efforts to decouple economic opportunity from the proximity to flood hazards (Di Baldassarre et al., 2019). For example, the provision of low-cost transport may allow people to access jobs while living further afield, potentially reducing the need to live in hazardous locations (Dodman et al., 2017). One course of action associated with these activities is to relocate existing informal settlements to the newly developed land (Johnson, 2018). However, despite the ostensible parsimony of relocation and resettlement schemes, several documented policy cases show the importance of ensuring that migration away from the floodplain is a carefully managed process which does not inadvertently increase poverty and inequality (Jain & Bazaz, 2020). Forcible evictions, which are commonly associated with the “high efficiency, low equity” paradigm described above (e.g., Amoako, 2016; Watson, 2014), violate human dignity and may not work as intended (Amoako & Inkoom, 2018; Jain & Bazaz, 2020). The failure of many such programs emphasizes the interconnected nature of urban systems and the myriad factors which determine flood risk (Amoako et al., 2019), leading many urban development experts to suggest that these policies should be considered only as a last resort (Jain & Bazaz, 2020; Johnson, 2018; Satterthwaite, 2001). Overall, it is imperative to develop risk management policies in partnership with communities (Mitra et al., 2017), which is identified as a hallmark of the “high efficiency, high equity” ideal to which the SDGs and the Sendai Framework aspire (Pelling, 2020). The insecure tenure status of many informal settlements is a key impediment to meaningful dialogue with city authorities (Dodman et al., 2017; Mulligan et al., 2016), leading Olthuis et al. (2015) to argue that an essential step toward improving the lives of inhabitants of informal settlements is to accept informal settlements as a permanent feature of the urban landscape.

The difference in outcomes between the two high equity scenarios shows that economic prosperity is vital to ensure recovery from natural hazards is sustainable. However, as the “high efficiency, low equity” scenario demonstrates, prosperity in one section of society does not necessarily translate to prosperity in other sections. This illustrates the need for policies which actively aim to reduce social inequality across the various facets of flood risk. Flood risk reduction requires long-term planning, coordination, and investment at various levels of governance, and the involvement of civil society (Di Baldassarre et al., 2014), all of which are severely hampered by structural inequality (Mitra et al., 2017). In the scenarios without wealth redistribution the existence of the unplanned settlement is tenuous and frequently enters a period of long-term decline. This is consistent with the vicious cycle identified by Islam and Winkel (2017), whereby persistent flooding disproportionately impacts poorer residents and deepens inequality. However, wealth redistribution on its own is not enough: there is also a need to encourage sustainable economic growth across society. This is shown by the results for the “low efficiency, high equity” scenario (Figure 7), where despite higher levels of equity the inefficient use of available resources means that both planned and unplanned suffer economic decline as a result of repeated flood events. While undoubtedly the private sector has a critical role to play in driving economic growth (Watson, 2014), it is important that appropriate governance structures are in place to ensure that private-sector led development is equitable, both in terms of the development process (e.g., purchasing of land, gaining planning permission, construction), and outcomes (Pelling, 2020). Whether this can be achieved within the prevailing neoliberal system is the subject of much debate (Gillespie, 2016).

The model we have developed has some important limitations. It currently treats the vulnerability and threshold of tolerable risk of the communities as static properties, whereas in reality both characteristics may vary in relation to other state variables. Furthermore, the model does not account for the effect of conflict on flood risk. As we have mentioned previously, many informal communities live in conflict with the city authorities and formal communities (van Voorst, 2016), which may arise because of the threat of forcible evictions from hazardous areas, or a political tradition of blaming informal communities for increasing the flood hazard (van Voorst, 2015). Similarly, we do not consider the role of democratic accountability in determining flood risk management strategies. This is highly relevant to informal settlements, where residents may be disenfranchized, or targeted by populist policies which rarely come to fruition (e.g., Amoako & Frimpong Boamah, 2015), eroding confidence in the social contract (Mitra et al., 2017). Here, social capital theory may provide a useful framework for including these facets of disaster resilience (Aldrich, 2012). Furthermore, there is a need for models to consider the role of institutions in shaping urban flood risk and enacting flood risk management strategies (Brelsford et al., 2020). We echo previous calls for socio-hydrological researchers to engage with social scientists to include these important processes in our models (Brelsford et al., 2020; Di Baldassarre et al., 2019). Another potential development would be to include the provision of foreign aid to disaster risk reduction efforts, which often underpins investment in risk reduction infrastructure in developing countries (Dodman et al., 2017). As Pelling (2020) note, the “low efficiency, high equity” scenario is often associated with disaster risk management and recovery which is led by aid agencies, since these organizations often lack the capacity and long-term outlook to stimulate sustainable economic development. Addressing these limitations will enable a more complete understanding of the dynamic relationship between urban flood risk management and social inequality.

Despite its shortcomings, our model lays the groundwork for more complex models which could provide a more detailed representation of coupled human-flood systems. As neither planned nor unplanned settlements are monolithic, further work needs to consider the heterogeneities which exist within these communities. These may present over the spatial dimension, but could also exist along lines of gender, age, and disability, for example. One possible approach to incorporate additional complexity is to use agent-based modeling, which would allow a more nuanced representation of the interactions between various actors within the system. The explicit representation of space in agent-based models would enable a better representation of floodplain hydrology, permitting a more rigorous assessment of the important spatial effects, such as the degree of co-benefits experienced from flood protection or, conversely, the transfer of flood risk from one area to another as a result of structural measures (Douglas, 2017). However, given their significant data requirements, parameterizing this class of model is non-trivial (Blair & Buytaert, 2016), reinforcing the importance of collecting relevant data on physical and socioeconomic phenomena to support future socio-hydrological research.

Urban socio-hydrology takes a water-centred view of risk which, to date, has tended to focus on large flood events (Di Baldassarre et al., 2013). This represents a weakness of applying socio-hydrology to understand risk in cities, where hazards exist on a spectrum, from everyday hazards, small disasters, and catastrophic events (Adelekan et al., 2015). It is often those living informally who are most vulnerable to small-scale events including localized floods, water shortages, infectious diseases, building collapse, fires and road traffic accidents, among many others (Dodman et al., 2017). The agglomeration of interrelated risks in urban areas demands policy responses that exploit opportunities for synergies and coherence across sectors (Adelekan et al., 2015; Mitra et al., 2017), as espoused by SDG Target 17.14, to “enhance policy coherence for sustainable development” (United Nations, 2015). By taking a holistic view of coupled human-natural systems, socio-hydrology is well placed to identify these opportunities. Toward this goal, we make three suggestions for future socio-hydrological research to make a meaningful contribution to the Sustainable Development Goals. First, research must recognize social inequality as a fundamental concern of sustainable development, and seek to increase knowledge about the way that human-water systems may influence social justice. As we have shown, reducing inequality in cities requires proactive measures which acknowledge the potential of urban development to establish or reinforce vicious cycles that exacerbate poverty. We also suggest that research efforts on water-related risks in cities need to be placed in the broader context of urban disaster risk reduction and urban development. Cities are places of multiple interacting hazards (Dodman et al., 2017), as well as centers which which provide myriad opportunities for addressing environmental and social challenges (Dodman et al., 2017; Ezzati et al., 2018). Lastly, recognizing that urban flooding and other water crises arise from failures of governance, future work on urban flood risk management should aim to contribute to discourse about reforms to urban governance and planning systems for urban disaster risk reduction and sustainable development.

## 7 Conclusion

In this study, we have put forward a system dynamics model to simulate interactions and feedbacks between urban flood risk management and social inequality in rapidly urbanizing cities. Our analysis demonstrates the importance of understanding social heterogeneities in managing urban flood risk, and highlights the potential for unforeseen policy outcomes which exacerbate inequality and poverty. Two overlapping policy approaches for successfully managing urban flood risk are apparent. First, our model supports the notion that reducing the vulnerability of informal communities through measures associated with slum upgrading projects can in some cases reduce flood risk to a level which is tolerable to the residents. Examples from cities around the world show that accepting the permanence of informal settlements and, in doing so, providing secure tenure to residents is an essential part of this approach. At the same time, our analysis suggests that city authorities must take proactive measures to ensure that future urbanization occurs on less hazardous land, taking into account the social and economic needs of future residents. While in some cases this approach may precipitate the relocation of existing informal settlements located in areas perceived to be high risk, such efforts should only be done in consultation with the residents of informal communities.

In line with previous socio-hydrological models, our model is a mathematical representation of idealized facts which has been developed to examine the interactions and feedbacks between system components (Di Baldassarre et al., 2013, 2015). Further model development is needed to include some potentially important processes and feedbacks in coupled human-flood systems which are relevant to social inequality. An outstanding task is to promote the static parameters representing vulnerability and risk tolerance to dynamic state variables. Additional complexity may be included by integrating our model into an agent-based modeling framework. Nevertheless, our work provides an important first step toward a quantitative understanding of the role of urban flood risk management on social inequality. Our results show that different flood risk management approaches can have large positive and negative impacts on marginalized communities, reinforcing the need for research which seeks to understand the dynamic relationship between water management and inclusive sustainable development.

## Figures and Tables

**Table 1:** Four Idealized Scenarios of Flood Risk Management, Adapted From Scenarios of Disaster Risk Recovery Outlined By Pelling (2020)

Scenario	Description	Examples
High efficiency, low equity	An approach to risk management which prioritizes the needs of the private sector. Reconstruction is efficient, consisting of risk management projects which aim to protect high-value assets and raise the economic value of the land (Pelling, 2020). Such projects fail to sustain or enhance social equality, either because they do not raise the protection level for the urban poor or because, once the protection is installed, urban authorities force residents to relocate in order to use the vacated land for high-value economic activity.	Buenos Aires, Argentina (Ríos, 2015) Kolkata, India (Rumbach, 2014)
Low efficiency, low equity	Characteristic of risk management in chaotic situations, such as might be seen in conflict-ridden and fragile societies, or societies which are extremely poor (Pelling, 2020). It may also describe the situtation where risk governance institutions are fragmented and under-resourced, leading to unclear roles and responsibilities and inefficient risk management. The result is a vicious cycle which increases the vulnerability of the urban poor to a variety of hazards.	Niamey, Niger (Boubacar et al., 2017) Accra, Ghana (Amoako et al., 2019) Cape Town, South Africa (Ziervogel et al., 2016)
Low efficiency, high equity	Risk management which prioritizes equity without properly considering how to build a sustainable economic recovery following disasters. As Pelling (2020) points out, this is a common outcome when the recovery is led by the humanitarian sector, which generally commits to ensuring equitable access to its resources during recovery yet lacks the capacity and long-term outlook to initiate a sustainable economic recovery.	Blantyre, Malawi (Hendriks & Boersma, 2019)
High efficiency, high equity	The stated goal of both the Sustainable Development Goals and the Sendai Framework: disaster risk management which enables equitable and inclusive sustainable development.	Nairobi, Kenya (Mitra et al., 2017)

*Notes*. Examples of each scenario are given. It is important to bear in mind that actual approaches to flood risk management are unique and may include elements of each of the four approaches, as well as features not covered by them.

**Table 2:** Parameter Values for the Four System Archetypes: (a) High Efficiency, High Equity; (b) High Efficiency, Low Equity; (c) Low Efficiency, High Equity; (d) Low Efficiency, Low Equity

	Description	High efficiency		Low efficiency	
		High equity	Low equity	High equity	Low equity
*ξ_H_*	Proportion of additional high water due to flood protection	0.5	0.5	0.5	0.5
*α_H_*	Inversely related to the vulnerability of human settlement	10	10/5.2	10	10/5.2
*ρ_E_*	Maximum relative growth rate, scaled by rate of flooding	1	1	0.5	0.5
*γ_E_*	Cost for unit height *R* and width $\sqrt{G}$ of flood protection infrastructure	0.005	0.005	0.005	0.005
*λ_P_*	Distance at which people would choose to live bearing in mind previous catastrophic flooding, scaled by the distance of no growth	2/0.666	2/0.666	2/0.666	2/0.666
*σ_P_*	Rate at which new properties can be built	0.1	0.1	0.1	0.1
*ϵ_T_*	Safety factor for flood protection	1.1	1.1/0	1.1	1.1/0
*κ_T_*	Rate of decay of flood protection	0.1	0.1	0.1	0.1
*α_S_*	Proportion of shock after flooding if protection installed (community)	0.5	0.5	0.5	0.5
*α_S_*′	Proportion of shock after flooding if protection installed (society)	0.5	0.5	0.5	0.5
*μ_S_*	Memory loss rate (community)	0.25	0.25	0.25	0.25
*μ_S_*′	Memory loss rate (society)	10	10	2	2
*τ*	Level of wealth redistribution	0.5	0	0.5	0

*Notes*. Parameters which are the same across formal and informal settlements are shown as single values, while parameters which vary between formal and informal settlements are shown as formal/informal.

## Data Availability

The model implementation and data are available online (http://doi.org/10.5281/zenodo.4085200).

## Key Points

- The distribution of flood risk in urban areas is uneven, with the negative impacts experienced disproportionately by the urban poor
- Our model shows that reducing the vulnerability of informal residents to flooding can reduce inequality, even when their exposure is high
- Based on the model results, we argue that urban flood risk management should be rooted in pro-poor urban governance and planning agendas
